# Probing the Electrophysiology of the Developing Heart

**DOI:** 10.3390/jcdd3010010

**Published:** 2016-03-22

**Authors:** Michiko Watanabe, Andrew M. Rollins, Luis Polo-Parada, Pei Ma, Shi Gu, Michael W. Jenkins

**Affiliations:** 1 Department of Pediatrics, School of Medicine, Case Western Reserve University, Cleveland, OH 44106, USA; mwj5@case.edu; 2 Rainbow Babies and Children’s Hospital, Case Western Reserve University, Cleveland, OH 44106, USA; 3 Department of Biomedical Engineering, Case Western Reserve University, Cleveland, OH 44106, USA; rollins@case.edu (A.M.R.); pxm261@case.edu (P.M.); shi.gu@case.edu (S.G.); 4 Department of Medical Pharmacology and Physiology, University of Missouri, Columbia, MO 65201, USA; poloparadal@missouri.edu (L.P.-P.); 5 Dalton Cardiovascular Research Center, University of Missouri, Columbia, MO 65201, USA

**Keywords:** cardiovascular development, cardiac conduction system, electrophysiology, electrodes, optical mapping, optical pacing, atrioventricular junction, sinoatrial node, atrioventricular node, His-Purkinje system

## Abstract

Many diseases that result in dysfunction and dysmorphology of the heart originate in the embryo. However, the embryonic heart presents a challenging subject for study: especially challenging is its electrophysiology. Electrophysiological maturation of the embryonic heart without disturbing its physiological function requires the creation and deployment of novel technologies along with the use of classical techniques on a range of animal models. Each tool has its strengths and limitations and has contributed to making key discoveries to expand our understanding of cardiac development. Further progress in understanding the mechanisms that regulate the normal and abnormal development of the electrophysiology of the heart requires integration of this functional information with the more extensively elucidated structural and molecular changes.

## 1. Introduction

### 1.1. Why Is It Important to Study Electrophysiology of the Developing Heart?

The heart begins to beat at a very early stage in its development when it is still a “simple” tube [1,2,3]. While embryos can survive to an early looping heart stage without a heartbeat [4,5,6,7], its function is critical for normal development and survival [8].

**Figure 1:**
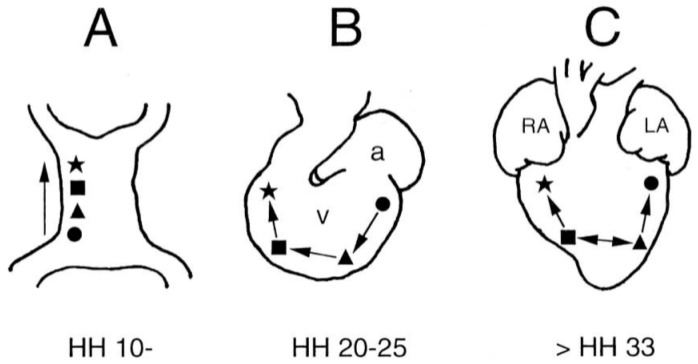
Activation sequence transitions. Activation sequence of the developing heart from tubular stages (**A**), looping stages (**B**), and after completion of septation to become 4-chambered (**C**). The activation sites represent the LV base (circle, ●), LV apex (triangle, ▲), RV apex (square, ■) and RV base (star, ★). a=atrium, v=ventricle, RA = right atrium and LA=left atrium. From [14].

The heartbeat even at the tubular heart stages has a global influence. The spontaneous electrical activation and coordinated impulse conduction of the tubular heart regulates the contraction of the heart that controls blood flow that in turn regulates the subsequent development of the heart and the embryo [9], blood cell formation [10,11,12], and likely the development of the extraembryonic vasculature including the yolk sac and placenta [13]. Each phase of embryonic development is matched by different patterns of activation and contraction of the heart (Figure 1). How these patterns are developed and how they go awry during abnormal development or disease are important to understand for early diagnosis/detection of congenital heart defects (CHDs) and other congenital defects and to reveal mechanisms that could guide us to strategies for accurate diagnosis, prevention and/or therapy. 

A potential value of this information would be in the synthesis of pacemakers or parts of the cardiac conduction system (CCS) *in vitro* so that they could be implanted into the diseased or damaged heart [15,16]. Another strategy that is being explored is to induce local cells to acquire properties of the pacemaker or parts of the CCS to replace the function of diseased tissues, e.g., [17,18,19,20]. Another challenging approach would be to develop and apply technologies to alter cardiac function of the improperly developing embryonic heart to prevent or alleviate downstream consequences.

### 1.2. What Are the Barriers to Studying the Electrophysiology of the Developing Heart?

The embryonic heart begins to beat when it is very tiny, approximately 100 microns wide (at narrowest point) and 300 microns long in a chicken embryo (around stages 9–10, 6–7 somite stage in chicken embryos, [21]). The mouse embryo heart starts to beat between stages E8–9 (equivalent to Carnegie stage 10, human post-conception days 22–23) and an E9 heart is 300–500 microns wide [22]. The small size of the embryonic heart is itself a barrier to study requiring high resolution detection. At these stages the embryonic tissues including the cardiac tissues are very soft and fragile which is another barrier to its study. Any mechanical intervention or contact may harm the cardiac tissues or alter impulses. Another complexity is the constantly changing topology of the developing heart. Accessing all surfaces for a global analysis of heart conduction would be difficult. Yet another challenge is the small electrical impulses emanating from such tiny hearts that require detectors with high sensitivity and enhancements of the signal to noise ratio. Analysis of the constantly beating heart will also require high speed recorders for thorough analysis.

Overcoming these multiple barriers to study the electrophysiology of the developing heart requires the development and application of new technology as well as the use of various animal models including non-mammalian models (see Section 1.4).

### 1.3. Overview of the Development of Pacemaking and Conduction Patterns of the Heart

Development of the pacemaking and conduction system has been previously reviewed [23,24,25] and will be briefly explained here with the addition of some newer findings. Stages of chicken embryogenesis as described by Hamburger and Hamilton [26] will be indicated as HH stage x. Equivalent mouse and human stages are indicated in parentheses in some places. Quail embryo stages are similar to chicken in the early stages (stage 4–28) [27] so Hamburger and Hamilton staging criteria will be used for them. 

#### 1.3.1. Earliest Stages of Cardiac Development

In chicken embryos, the precursors of heart cells appear between HH stage 5–6 as cells coalesce in the anterior lateral plate mesoderm on the left and right and fuse to form a crescent by HH stage 9 [28]. These cells begin to express markers such as *Nkx2.5* and *Isl1* and form a tubular structure [29,30,31,32]. The heart begins to beat when it is still a tube and before looping begins. The pacemaking region was identified by detecting which cardiomyocytes contracted or activated first during the heart cycle [21]. More recently the precursors of the mature pacemaker have been found to be outside the identified 1st and 2nd heart fields suggesting that the initial pacemaker region and the later appearing pacemaker regions are distinct [33]. The tubular heart exhibits slow and homogeneous conduction from the caudal to cranial regions as determined by observing contraction and optical mapping (OM) [3,34,35,36]. The tubular heart cardiomyocytes are thought to be capable of spontaneous activation because when dissociated, many can still contract as single cells [37]. Why a particular small group of cells in a particular region of the heart tube control the heartbeat is not certain. 

#### 1.3.2. Alternating Regions of Slow and Fast Conduction of the Looping Heart

The second pattern of cardiac conduction that emerges is alternating regions of faster and slower conduction. The atrial and ventricular myocardium conducts rapidly while the sinoatrial (SA), atrioventricular junction (AVJ) and outflow tract (OFT) myocardial regions conduct more slowly [38]. The histology, molecular expression, and the action potential (AP) of the AVJ was also reported to become quite different from those of the flanking atrial and ventricular myocardium [2,39,40,41,42]. Some of these differences have been proposed to be the result of the distinct composition and size of the extracellular matrix adjacent to these myocardial regions [43,44]. The fast conduction within the atrial and ventricular chambers allows near simultaneous contraction within these chambers, potentially elevating the forward blood pressure to achieve higher flow velocity. The slow conduction at the AVJ and OFT allows discrete and sequential contraction of the atrial chamber followed by the ventricular chamber, preventing retrograde blood flow by prolonged closure of the slower conducting sections of the heart tube. This pattern still allows the base to apex direction of conduction within the ventricle. 

The mechanisms that drive development of pacemaking tissues and portions of the cardiac conduction system are still far from understood and controversial [45,46,47]. The AV canal or AV junction in the embryonic heart appears to serve the function of the AV node in the adult, that is, to slow conduction to allow the atrium to activate and contract fully before the ventricle. Exactly how and what part of the AV canal myocardium transitions into the mature AV node is still unknown. The complex anatomy, electrophysiology and molecular expression of the adult AV node [48,49,50,51] suggests that different tissues and cell types may come together during development to create the final product. Endothelin, Notch and Wnt signaling among the cell types at the AV junction are all involved in AV canal differentiation and impact the electrophysiology of the cardiomyocytes of the AVJ [43,52,53]. Certain sections of the AVJ are removed by apoptosis and replaced by connective tissue components [54]. However, information regarding the next steps in maturation of the AV junction and/or node remains incomplete.

#### 1.3.3. Transition to the Apex-to-Base Conduction

The third pattern that appears over several stages is that of the mature conduction system in which the electrical impulse travels rapidly from the sinoatrial (SA) node through the atrial myocardium to the atrioventricular (AV) node where the impulse is delayed before it is conducted by the central ventricular conduction system (the common bundle also termed the Bundle of His and the left and right bundles) and its terminal fibers (Purkinje fiber system) to the apex of the ventricle where activation of the more apical ventricular cardiomyocytes occurs first followed by a spread of activation towards the base of the ventricles and finally to the outflow tract. The emergence of this pattern involves the maturation of the cardiomyocytes but also non-cardiomyocyte cells and tissues at the AVJ that create the fibrous AV ring. These latter cells, that include derivatives from the endocardium, epicardium, and neural crest, insulate the atrium from the ventricle except at the common (His) bundle [54,55]. Insulation of the central cardiac conduction system is also critical to allow the impulse to travel to the apex of the ventricle without activating adjacent working myocardial cells. This final mature pattern of conduction is critical for the coordinated contraction of the heart required for efficient movement of the blood through the heart and ejection into the circulation that supplies the organism and extraembryonic tissues. 

Some information is available about the mechanisms of maturation of the peripheral conduction system within the ventricle. These Purkinje cells appear to be recruited from working myocardium by endothelin signaling from the endothelium of coronary vessels and the endocardium [24,56,57,58,59,60,61,62,63]. The evidence suggests that the signaling may well be regulated by shear or other mechanical forces impinging on endothelial cells. Although endothelin 1 signaling was presumed to be critical for normal mouse heart development as for avian heart development, deletion of endothelin receptors in the mouse had no apparent effect on the structure or function of the conduction system [64]. Neuregulin and Notch signaling play important roles in conduction system development in the mouse model [53,65]. The relationship between all these signaling cascades and how they are triggered requires further study.

With these questions unanswered or with ambiguous or apparently contradictory answers, it is important to continue the study of the pacemaking and cardiac conduction system development. The creation, improvements, and application of technologies to assay cardiac function may help us to better understand cardiac development and test and integrate that information with structural information and proposed molecular and cellular mechanisms. 

### 1.4. Animal Models for the Study of Cardiac Function

Heart development has been largely elucidated by the study of animal models. While certain assays can provide information about the function of the developing human hearts during pregnancy, the limitations are many. The animal models commonly used to study heart development are fruit fly, zebrafish, mouse, and chicken or quail.

#### 1.4.1. The Fruit Fly

The larvae of the common fruit fly *Drosophila melanogaster* has a tubular heart termed the “dorsal vessel” that pumps hemolyph, the equivalent of blood, in both directions. It has been useful in the analysis of cardiogenesis mechanisms because its genome can be so easily manipulated [66]. An example of strides attributable to fruit fly research include the discovery of the *Tinman* gene that led to study of the homolog genes in humans and mice. As the name implies, the fly larvae with the mutant gene lacks a heart [67,68]. Since then, several homologous genes in humans were identified that when mutated were associated with heart defects [69]. Pacing the Drosophila heart at larval, pupal, and adult stages has been achieved using optogenetics with OCT (optical coherence tomography) used to capture the function [70]. Other examples are the discoveries that (1) the activity level of ORK1 (outward rectifying potassium channel 1) regulates the heartbeat [71] and (2) a 2-pore domain K^+^ channel of a TRPA channel (*Painless*) is critical for the response of the heart to mechanical constraints [72]. These latter findings used the combination of analyses of Drosophila mutants using standard electrode recordings and videomicroscopy. This animal model provides the opportunity to study in more detail the relationship of cardiac function and molecular changes *in vivo*. The obvious limitation of this insect model is that the final anatomy of this tubular heart does not resemble the four-chambered human heart. However, this model continues to provide critical information relevant to vertebrate development and human disease [66,73,74].

#### 1.4.2. Zebrafish

The zebrafish (*Danio rerio*) is highly conducive to genetic manipulation and resembles more closely than Drosophila the early stages of human heart development. The zebrafish develops chambers, an epicardium, coronaries and a conduction system but does not develop a four-chambered heart. The zebrafish embryo is transparent so its cardiac structure and function can be followed all the way to hatching. Zebrafish can also survive to 7 days post-fertilization despite severely compromised heart development and even with an absence of heart function [7] because the embryos can survive on diffused oxygen alone [75] without any blood circulation. This is a benefit because cardiac defects can develop long enough to become obvious and easier to detect and analyze but it is also a limitation because it does not reflect what happens in mammalian embryos that die much sooner (at early looping heart stages) without a heartbeat [4]. 

A particularly important strength of this model is its accessibility to genetic manipulation [76]. Forward genetic screens have identified a number of mutants that have cardiac defects with some having functional defects [7,77,78,79,80]. The “reverse” genetic approach, using anti-sense morpholino oligers termed morpholinos can also be used effectively to manipulate gene expression of zebrafish embryos by interfering with translation, splicing, and microRNA functions [81,82] with the caveat that off target effects have to be seriously considered in light of findings that morpholinos may frequently produce false positives compared to gene targeted methods [83,84]. Nonetheless, this accessible model is likely to be valuable for probing the electrophysiology of the human heart because it has similarities in ion channels and calcium-handling proteins [85].

#### 1.4.3. The Birds

The avian (chicken and quail) heart is very similar in overall structure and function to that of the human heart with some differences in detail. An example is that the chicken right atrioventricular valve has two leaflets while humans have three. Avian embryos are limited in genetic manipulation compared to the mouse or zebrafish but engineered viruses and electroporation can be used to change molecular expression. The use of lentiviruses has allowed the creation of transgenic quails with endothelial cells or neurons fluorescently marked [86,87,88,89]. A big benefit of the avian model is the long history of work on these animals and the accessibility of the embryo to assays and manipulations. The embryos can be imaged longitudinally to capture structure and function under near physiological conditions [90]. The neural crest ablation model was the first reliable model of CHDs in an experimental animal and has served as the “gold” standard for defining the pathogenesis of heart defects in other experimental models [91]. The consequences of deletion of the cardiac neural crest closely mimicked the DiGeorge/22q1.2 deletion syndrome in cardiac and glandular phenotypes thus pointing to dysfunction of neural crest as a mechanism for the syndromes [92,93,94,95].

#### 1.4.4. Mouse

The main advantage of using the mouse is it can be genetically engineered. Many engineered mice are available to study and continue to be generated at a rapid pace. It has been the model of choice in the analysis of transcription and other factors that might impact heart development. The developing mouse heart is very similar in phenotype to that of the human heart [96]. For functional studies however, the caveat is that there are important differences in the physiology of the mouse heart compared to that of the human heart [97,98,99].

Many markers for the pacemaking and conduction system have been discovered in this model [100,101]. A fortuitous finding was that the sinus node and CCS were marked in a mouse with a reporter gene inserted into the genome [102,103]. This and other mouse lines (see Table 1 of relevant mouse lines provided by Liang *et al.*, 2015 [101]) allow studies correlating function and anatomy in embryonic and adult hearts. The transient and complex expression patterns of some of these markers during development [101] suggest that it will be a challenge to understand their precise significance. It will also be a challenge to reproduce just the right gene expression patterns at the right time to induce proper conduction system differentiation *in vitro* or *in situ*.

#### 1.4.5. Rat

The rat has been widely used for research because it is a hardy and prolific species. While still limited for genetic manipulation, novel technologies such as CRISPR/cas9 may overcome that hurdle [111,112]. The cardiac conduction system in the embryo and adult rat heart has been studied histologically using various markers [113,114] but few studies have combined functional and anatomical assays on the developing heart of the rat.

#### 1.4.6. Rabbit

The rabbit model has been used extensively for the study of adult heart physiology. For developmental studies, the rabbit model is unique in that the pacemaking and conduction system can be labeled by antibodies directed to neurofilament protein 160 in the embryo and the adult [115,116] or by *in situ* hybridization to the mRNA for neurofilament M [117]. Despite this unique opportunity, few developmental studies have been conducted to take advantage of this model [117,118,119]. The difficulties in using this model are that rabbits cannot be easily genetically modified and they are expensive to use for large cohort studies.

#### 1.4.7. Large Animals

Larger animal models such as dogs, sheep, pigs, and goats have the advantage that they can be more easily instrumented and otherwise probed for *in utero* information, e.g., [120,121,122,123,124,125]. Their Physiology is more similar to humans than rodents. For some of these species, glycogen stores are clearly more robust in the conduction system and PAS (Periodic Acid Schiff) histological staining can delineate the Purkinje fibers [126,127,128] but these species have not been used extensively for developmental studies. These larger animals are not conducive to studies requiring large cohorts because of the expense and the space required for their maintenance. Another limitation is that genetic manipulation is difficult in these animals except with short term transfections. 

## 2. Electrodes and Application to Study Cardiogenesis

Electrical activation was originally deduced from the careful study of contraction patterns (e.g., [3,34]). Later, electrodes were used as the first probes to directly study electrophysiology of the heart. With the exception of the studies of van Mierop [129] who was able to use electrodes to study 8-somite (HH stage 9+) chicken embryos, many studies during the early years were necessarily conducted on larger avian embryos, those older than 3 days of incubation (HH stages > 20–23).

### 2.1. Electrodes and Technical Considerations

Microelectrodes, glass microelectrodes, micro-pipettes, and sharp electrodes were for many years the workhorses of developmental cardiac electrophysiology studies. Microelectrodes were introduced in 1946 by the American scientists R. Gerard and G. Ling to obtain the electrical potential of a mouse neural fiber and later, on a single cell [130]. A variant of this has been the use of metallic microelectrodes with tip diameters of ~1 micron. Glass micropipettes are made from glass capillary tubes often with an intratubal filament. Most of the glass used today is borosilicate; however, a quartz tubing for micropipettes is also used. Quartz micropipettes are less noisy than borosilicate, but require specialized and expensive (*i.e.* typically CO_2_ laser based) pullers. Pipettes normally are filled with a 3 M KCl solution and due to their small tip (usually <0.1 mm) present high impedance when compared with suction electrodes or patch clamp electrodes. A regular puller can be used to generate microneedles, micropipettes and patch pipettes. The main differences between pipettes are the dimensions of the glass tube used and the parameters used to pull the pipettes (heat, velocity and force of the pulling and cooling). Because of the high impedance characteristic of the microelectrodes for many years, the gold standard protocol for using these electrodes required a bridge amplifier. Until the last several years, multipurpose amplifiers (*i.e.*, used for patch clamp experiments) were ineffective to reproduce the characteristics of a bridge amplifier. The microelectrode morphology and use are defined mainly by the taper of the tip and the length of the tip. Changes in these parameters allow the manufacture of rigid or more flexible micropipettes. Longer tips have a larger resistance than smaller tips but they are more flexible. This flexibility is key to performing intracellular recordings in intact spontaneous beating hearts. However this presents a problem when recording from older or mature hearts because the consistency of the thicker and harder extracellular matrix surrounding the cells limits the access of the micropipette into the cardiomyocytes. On the other hand, a rigid tipped micropipette may easily impale cardiomyocytes in mature hearts but the contractions of the heart limit making continuous recordings from the same cell. Thus there is no one protocol suitable to obtain the best cardiac intracellular recordings for all occasions. The ideal parameters all depend on the size of the heart to be recorded, how the heart is immobilized, the angle used to impale the cells, and how fast the cells are impaled sometimes using an electronic BUZZ system or a tap on the recording apparatus. The BUZZ circuit facilitates cell membrane penetration by providing very powerful high frequency pipette tip oscillations caused by overcompensating the capacitance compensation of the system. One of the key advantages of intracellular recordings is that the cells “preserve” all their intracellular content and therefore could be operating under “normal conditions”. Typically recording from embryonic cardiomyocytes occurs over a couple of seconds to minutes. In rare occasions it is possible to maintain recordings from a single cell in an intact heart for several hours. Thus intracellular recording of an intact heart is more of an art than a science that develops with time and experience.

The unique beauty of the intracellular recordings in the heart is that it reveals the AP of single cells in the intact heart or in isolated cells. By analyzing the properties of the AP recorded (amplitude, maximal rate of rise, duration and general shape) it is possible to recognize the place of origin or identity of the recorded cell (*i.e.*, atria, ventricle, Purkinje fiber, pacemaker, outflow tract, *etc.*) and even the stage of development (age of the embryo) of the recorded cell. Recording single cells in isolation is not as easy as it sounds because the isolated cell is not cushioned by the presence of other cells/tissue and thus it is very easy to break the tip of the pipette when attempting to impale a single cell sitting on a rigid plastic culture dish. Another important factor that affects the ability to record cardiomyocytes from intact hearts, pieces of tissue, explants, or single cells is the condition of the cell. Cardiomyocytes are very energy demanding and very sensitive to anoxia and temperature changes. Recordings at lower than physiological temperatures (32–35 °C) may improve the chances to impale and keep a recording for longer periods of time up to hours with AP characteristics similar to those obtained at 37 °C. The caveat with this technique is that specific APs characteristics are altered, mainly duration, and frequency. 

Electrodes are particularly suited for the analysis of cultured cells. Creating single cell cultures of healthy cardiomyocytes was for many years very difficult. Today with the advances in dissociation techniques this limitation has been eliminated. By using a modified papain protocol [44] it is possible to produce cells in a healthy enough condition so that 99% can be successfully subjected to electrophysiological protocols. Another important consideration is that every embryonic isolated cardiomyocyte is a potential pacemaker. Thus isolation of cardiomyocytes from early stage hearts produces cultures in which the cells should be spontaneously beating up to 2–3 h after dissociation. Cell cultures made from older hearts (~>6–7 days old chick heart or the equivalent in the mouse) do not generally begin to beat until several days in culture. However, these can be stimulated to beat through the recording electrode.

Suctions electrodes, an alternative to microelectrodes, are characterized by a tube often made of polypropylene that is attached extracellularly to the cells by suction. The advantage of this type of recording tool is that the tip is very flexible and can follow the movement of the beating heart. However, in practice it is difficult if not impossible to record from a single cell because small leaks in the seal between the pipette and the cell result in alterations in the morphology of the AP recorded (typically the AP will also be smaller in amplitude). Another drawback to this technique is that it does not allow for a quantitative evaluation of some AP properties (*i.e.*, resting membrane potential and amplitude). The AP appears inverted compared to other methods.

Since the development of the patch clamp technique, a large number of scientists have used it to study cardiomyocytes. While the micropipettes allow for the recording of the AP in cells, patch clamping allows the characterization of the different ionic components of the AP, something that cannot be done with micropipettes alone. Some forms of patch clamping, like current clamping may allow the recording of the AP. A caveat with this technique is that, under the whole cell mode it is likely that all the intracellular components of the cell have been dialyzed with the content of the microelectrode solution. As a consequence the AP does not necessarily represent what is normally present in the cell. Only under current clamp in perforated patch mode is the recorded AP similar to those obtained by intracellular recordings.

Capturing electrocardiograms (ECGs) post-natally is usually accomplished by placing electrodes at specific sites on the torso and limbs. Capturing ECGs from embryos usually requires the removal of the heart from the embryo [131], although a recent report indicates that ECGs can be collected from intact Drosophila pupae using special electrodes composed of a novel liquid metal [132]. Magnetocardiography is used as a clinical tool for the analysis of fetal heart function showing the complex relationship between the maternal and fetal heartbeat [133,134]. Micro-magnetocardiography has been performed on adult rodents, [135,136] but has not been used to detect prenatal heart function in animal models. The expense of this method deters its use. 

The use of arrays of electrodes takes advantage of the benefits of electrodes while capturing data simultaneously from many points on a culture dish or tissue. These microelectrode arrays (MEAs) can also be used to deliver impulses. MEAs have been used extensively to study the cell sheets grown in culture. When mounted on stretchable/flexible substrates, they have also allowed mapping of various parameters including APs over the contour of adult hearts [137]. The use of MEAs for therapy has just begun, and its use has not yet been extensive in developmental systems.

### 2.2. Probing the Early Stages with Electrodes

**Figure 2:**
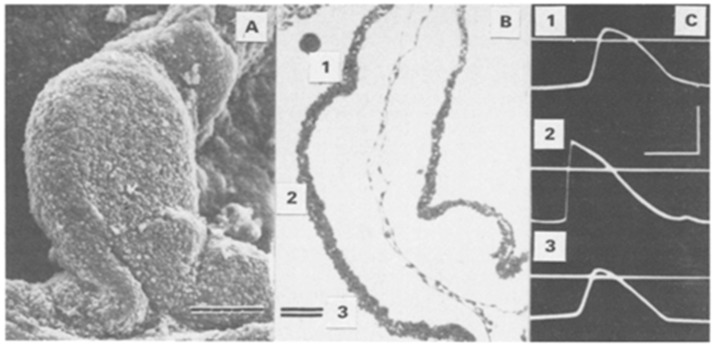
Early heart action potentials. Action potentials captured by electrodes placed on the early embryonic chicken heart [39]. (**A**) A scanning electron micrograph of the tubular heart (bar = 100 m) at 35 h (around stage 10); (**B**) Histological section of the same stage heart showing the myocardial wall to be two cells thick with a cardiac jelly separating it from the endocardial epithelium; (**C**) Resting membrane potentials (scale bars are 100 ms in time) and action potentials (APs) are 20 mV from the sites (1–3) labeled in panel B. At 40 h (stage 11) there is a change in AP morphology at site 2 showing a faster upstroke and a higher amplitude.

The earliest stage of heart development at which intracellular electrical activity was recorded is 35 h of incubation in a chicken embryo (HH stage 10 in chicken embryos is equivalent to embryonic day 8.5 in mouse and 24–26 days in human as determined by [26,138]). At this stage the fusion of heart tissues to become a tube is almost complete with a largely uniform two-cell thick myocardial layer [2,39,139]. At this stage the heart could be considered a tubular heart although far from a simple tube in structure and certainly already asymmetric [140,141]. The myocardium at this stage lies on the abundant extracellular matrix (ECM) of cardiac jelly of variable thickness that separates it from the endocardium. The resting membrane potential (RMP) of this myocardium was found to be −15 mV and the action potential (AP) was approximately 20 mV with a slow rising phase and a long duration (210–220 msec) [39] (Figure 2). An AP with a RMP of −15 mV and an amplitude of 20 mV is lower than we would expect based on what we know about the properties of K^+^, Na^+^ and Ca^2+^ channels in mature cardiac muscle cells. These puzzling findings may have an explanation in the properties of the very young cardiomyocytes.

Studies of the early electrical activity of the heart in the 1970s and early 1980s showed that cardiomyocytes exhibit small patterns of electrical activity before they contract [35,36,142,143,144,145,146,147,148,149,150,151]. The TTX receptor and the fast Na^+^ channel machinery have been shown to exist even when APs are insensitive to TTX (3 day old embryos, HH stage 20) [152]. The channel is then in a nonfunctional or silent form that is only revealed (or chemically activated) by both the alkaloids (veratridine and BTX) and the polypeptide toxin (ATX11) [152].

It has also been shown that the total number of Na^+^ channels increases during development by a factor of 4 or 5 (for review see [151,152]). It appears that there are few fast Na^+^ channels in young hearts. Therefore, in the early stages of development, the inward current of the AP is carried predominantly through TTX-insensitive slow Na^+^ channels. The early cardiac AP is carried mainly by calcium channels. We do not know the exact channel and the composition of the transporter in very young cardiomyocytes (<40 h, stage 11). The reason for the unusual electrical activity (small AP amplitude and resting membrane potential) at this stage remain elusive.

### 2.3. Alternating Regions of Slow and Fast Conduction in the Looping Heart

The emergence of the slow and fast conduction regions was documented using individual electrodes and electrodes glued together [38]. When the bulboventricular loop begins to form, between 39 and 42 h (HH stages 10–11), the AP of the cephalic and caudal portion of the looping heart exhibit similar shapes, with RMPs of −17 mV to −20 mV and amplitudes of ~30 mV [39]. At 45 h (HH stage 11–12), the posterior region of the heart begins to protrude and this newly-differentiated atrial region begins to exhibit a larger RMP of −28 mV and AP amplitudes of 45 mV, with a faster rising phase of shorter duration (~87 ms). The upstroke of this atrial AP is preceded by a slow diastolic depolarization, which gradually disappears at more advanced stages of development (72–90 h, HH stage 19–23), when the atrial cells begin to be activated by the pacemaker in the sinus venosus [39,40].

An incipient narrowing region (75–100 microns) between the atrial protuberance and the ventricular loop appears simultaneous with the formation of the atrium [39,153]. APs from this region showed a RMP of −22 mV and a slow rising phase, with an amplitude of 23 mV and a duration of ~180 ms. This area is known as the atrioventricular canal (AVC). In contrast, the ventricular cells had an RMP of −43 mV and an AP with amplitude of 58 mV and duration of 145 ms. At 62 to 67 h (HH stage 17–19), significant growth of both the atrium and ventricle render the AVC more evident and show the presence of the incipient cardiac endocardial cushions, the primordia of the valves [39,40].

The electrical properties of the different structures of the heart gradually change during these early stages of development. The ventricle is the first to differentiate, followed by the AVC and the atrium. The slow and small AP of cardiac cells at early stages is modified so that the RMP, the rate of rise and amplitude of the AP increases during development. The rate of rise is always greatest in the ventricular cells, followed by that in the atrial cells, and finally by that in the AVC cells. These observations suggest that the AVC is responsible for the AV delay before the appearance of the specialized conduction system. This AV delay causes the out-of-phase contractions of the atrium and the ventricle that create a gradient in pressure between the two chambers and thereby improve blood flow during the earliest stages of development, as much as 6 days prior to the appearance of the specialized conduction systems which appears at 7–10 days (HH stage 31–36) of development in the chicken embryo. 

In the early stages of heart development, electrical impulses originate in atrial cells, and propagate to the ventricle(s) through the AVC. The cells from the AVC, besides being responsible for the AV delay, generate slow and long-lasting APs. At this stage of development the AVC provides the 70 ms delay between the contraction of the atria and the ventricle for proper heart function. During the early stages of heart development, every cardiomyocyte is a potential pacemaker can generate spontaneous APs and contractions. When two or more of this cardiomyocytes contact, the one with the highest frequency seems to drive and/or synchronizes the rest of the cells in contact [154,155]. Thus during normal development, the cells with the highest frequency are likely to be the cells of the atria, especially the ones localized in the right atrium. 

**Figure 3:**
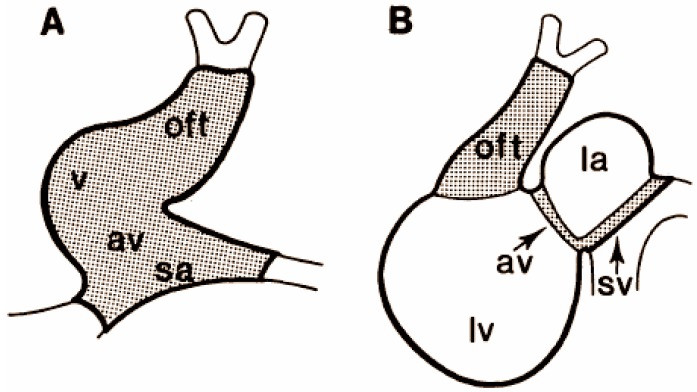
Slow and fast conducting regions. Alternating regions of slow and fast conduction in the looping heart of the chicken embryo detected using electrodes [38]. (**A**) The conduction velocity of the HH stage 13 (embryonic day 2) early looping heart was found to be slow and homogeneous; (**B**) This pattern changed to regions of fast conduction (white regions) alternating with regions of slow conduction (dotted regions) by HH stage 23 (embryonic day 4). oft = outflow tract, v = ventricle, av = atrioventricular junction, sa = sinoatrial junction, la = left atrium, lv = left ventricle, sv = sinus venosus.

In the study by de Jong *et al.*, extracellular platinum electrodes (25 μm in diameter) were placed on the heart surface at various places [38]. Some hearts were recorded during spontaneous beating and others were paced from the atrium or sinus venosus with a reference electrode on the ventricle. In some of their experiments electrodes were glued together with 2–3 terminals that were 40–170 μm apart and placed on various locations and angles on the heart surface. A current was applied after recording to burn and “mark” the recording sites that could be histologically detected. The data were used to analyze conduction velocity that was slow and remained relatively slow in the outflow tract from stages of looping to septation (0.5 to 1.3 cm/s at 3–7 day, HH stage 20–31) while the ventricle rapidly increased conduction velocity between those same stages (0.9 to 20 cm/s at 2–7 day, HH stage 12–31). A map of slow and fast conduction regions was created using this method (Figure 3) and revealed that these regions seem to correspond to maps created using molecular markers [156]. This supported the possibility that these functional differences reflected a unique molecular program of differentiation for these alternating regions. A hypothesis arose from these and other findings that the sinoatrial (SA) and AV junctions and the OFT retained their “primitive” tubular heart properties while the atrium and ventricle proceeded on a very different trajectory of growth and differentiation. A scenario of transcriptional activation, repression, and competition among several genes including GATA, Nkx2.5, Tbx5/2 and 3 has been proposed to explain the regulation of specific chamber genes including Cx40 (reviewed in Christoffels *et al.*, 2004 [41]). Potential limitations of this electrode-based method were that the direction of conduction was not easy to determine and the distances between sites, used to determine conduction velocity, may have been difficult to ascertain on irregular surfaces.

### 2.4. Transition during Septation of the Ventricle

The ventricle that has been activated from base-to-apex, transitions to apex-to-base. This transition has been attributed to two important changes, the maturation of the AVJ in maintaining slower conduction and becoming insulated from AV conduction at sites save where the common bundle crosses the AVJ at the dorsal aspect of the heart, and the maturation of the ventricular conduction system. In chicken embryo hearts, this transition was first reported to occur between HH stages 28 and 32 and discovered by the use of electrodes [14] and subsequently by the use of OM [157,158,159] as described below.

### 2.5. Maturation of the Pacemaking and Cardiac Conduction System

Maturation of the cardiac conduction after transitioning to the apex-to-base activation pattern occurs throughout the late fetal and postnatal stages. These steps appear to require the insulation of the central ventricular conduction system by connective tissue [159] and the maturation of the fibrous ring or annulus at the AVJ to separate the atrial from ventricular myocardium [55]. The final mature structure of the pacemaking and conduction system has been extensively revealed by Tawara and others [50,51,55,113,160,161]. The contribution of non-cardiomyocyte cells including neural crest cell progenitors and epicardially derived cells [159,162,163,164,165] as well as the importance of cell death [166,167] to the sculpting of the final mature structures of the central cardiac conduction system has been reported. However, there still remain uncertainties and controversies regarding the origins and maturation of the parts of the SA node and the AV node and the AVJ fibrous ring that will require further studies to clarify.

In the adult heart the functional organization of the conductive pathways is complex. The electrical activity is originated by pacemaker cells localized in the sinoatrial node (SA node) located in the upper part of the right atrium. Signals arising from the SA node stimulate the atria and travel to the atrioventricular node (AV node) at the interatrial septum. The AV node cells generate small and slow APs [168,169,170] and have few cell-cell junctions [171]. These and other unique characteristics of the AV node cells are responsible for the delay in the propagation of the electrical activity. After this delay, the stimulus diverges and is conducted through the left and right bundle of His to the respective Purkinje fibers that branch into and spread over each side of the heart from endocardial side of the myocardium at the apex of the heart and then finally to the epicardial side of the ventricular wall from the apex towards the base.

Molecular markers and mouse lines have provided some interesting and surprising clues regarding the origin and gene expression of the pacemaking and conduction system during development. HCN4 that is a marker for the SA node in the mature heart is expressed in the entire first heart field [108] and its gene expression is quite complex throughout development of the CCS. Parts of the SA node also express Tbx18 and at least some [172,173] of the progenitors surprisingly may come from the epicardium [172,173,174].

For many decades the embryological origin of the atrioventricular node (AV node) and bundle of His in mammals was confusing and attributed to different sources. The AV node was suggested to develop from the dorsal wall of the AVC at least in the mouse, rabbit, calf and human [175,176,177,178,179,180]. Other investigators considered that the AV node arose from the atrioventricular ring (AV ring) in humans [160,176,181,182]. In the 70s it was postulated that the AV node initiates from two primordia located in the dorsal wall of the atrium [171,183,184]. 

With regard to the origin of the bundle of His, this was more controversial. Some investigators proposed that the bundle of His developed from the interventricular septum [178,179,180]. Other groups considered that the bundle of His originated from the cells in the AV node [185,186,187]. More recently it was suggested that the cells from the dorsal wall of the AVC contributed to the formation of the bundle [171,183,184,188] while others proposed that the bundle derived from the bulboventricular or “primary” ring [160,182].

The earliest electrophysiological detection of the bundle of His cells and the presence of cells with characteristics of the AV node was between 5 1/2 and 6 days of chick development (HH stages 28 to 29) [40]. At this stage the bundle of His is not fully developed, lacking the right and left branches. The bundle of His cells were localized ventral to the AV node as a continuation of the same structure. These cells were localized in a circumscribed area located in the lowest and dorsal segment of the interatrial septum. Since there are no clear morphological difference between these groups of cells, only the shape of their AP allowed them to be identified [40,189]. Especially interesting is that these early AV node and bundle of His AP responses had similar characteristics to those observed in the adult heart [169,170,190,191].

### 2.6. Electrodes Summary

In conclusion, a range of electrodes can be used to assay cardiomyocyte parameters during development depending upon the kind of question that is being addressed. The higher temporal resolution of electrodes compared to other techniques makes it a preferred method for the detailed study of cells and ion channels. Even with more sophisticated multi-electrode arrays available, electrodes have many limitations for the study of conduction in the intact embryonic heart.

## 3. Optical Mapping and Its Applications

The features of optical mapping (OM) allow simultaneous analysis of important parameters such as conduction velocity and action potential duration over a large field of view. For that reason OM has been applied extensively in the study of the adult heart (reviewed in [192]). The challenge is to apply this method to tiny sensitive beating hearts that are undergoing constant and rapid morphogenesis.

### 3.1. What Is Optical Mapping?

**Figure 4:**
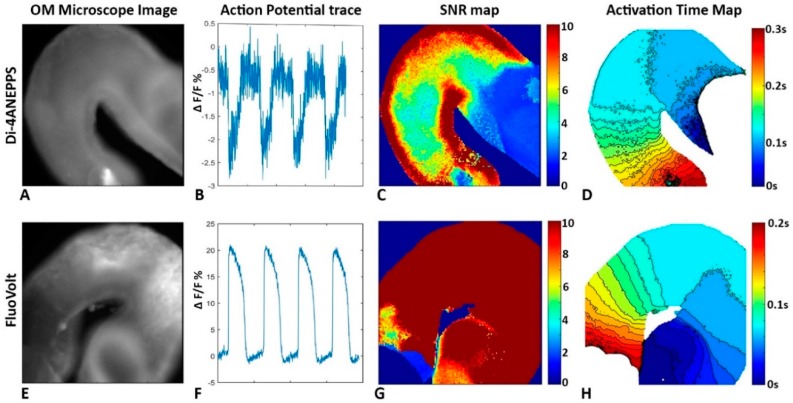
Isochrones calculated from optical mapping (OM) data. HH stage 15 quail embryo hearts were stained with Di-4-ANEPPS or FluoVolt^tm^ and subjected to OM to obtain (**A**) a microscope image, (**B**) AP traces, (**C**) SNR maps, and (**D**) 2-D activation isochrome maps. The use of FluoVolt (**E**) provided a cleaner AP trace (**F**), improved SNR over the heart (**G**), and isochrones maps (**H**).

**Figure 5:**
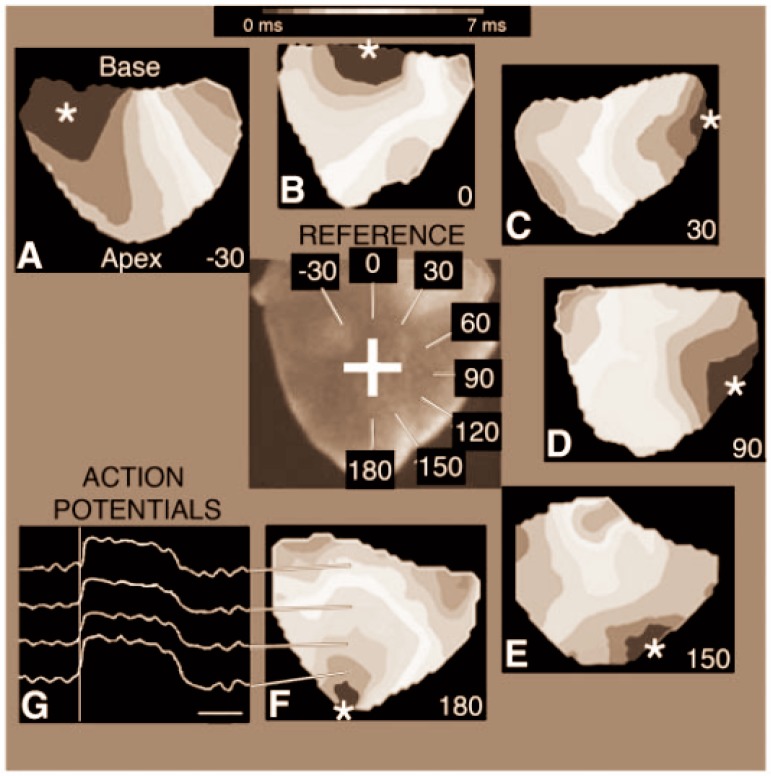
Transitions in ventricular conduction pattern detected by OM [159]. Isochrome maps and the angle value measurement system of ventricular activation were generated from the recordings of the posterior side of chicken hearts at HH stages 29–35. (**A**–**F**,**G**) Waveforms of optically records APs from several locations on the posterior side of the heart. “REFERENCE” indicates how angle values between −30 ° to 180 ° are assigned to isochrones map based on the site of the initial breakthrough of AP that is indicated by the asterisk (*). Bar = 20 ms in (**G**).

OM takes advantage of voltage sensitive fluorescent dyes (e.g., NK2367, Di-4-ANEPPS) that intercalate into the cell membrane and change emission spectra as the membrane potential changes. APs can be imaged using sensitive photodiode arrays or more recently fast CCD/CMOS cameras and analyzed to determine the activation sequence across tissues. These data allow creation of an isochrone map indicating the pattern of conduction throughout the heart (Figure 4 and Figure 5). The advantages of OM are that it requires no contact with the tissue during detection and enables the capture of data at high spatial resolution from a large field of view simultaneously.

Fast voltage sensitive dyes are able to track membrane potential changes with high temporal resolution. The kinetics of dye signals were characterized and compared to simultaneous electrode recordings as the gold standard [193,194,195]. These studies confirmed that optical signals recorded using the dyes have great linearity and correlation with electrode recordings.

The OM system requires an excitation light source, a fast photodetector and an appropriate filter set [196,197]. OM allows the imaging of transmembrane potentials over a large field of view and currently allows collection of hundreds of thousands of traces simultaneously with high spatial resolution [196,197]. Parameters in a single action potential such as activation time, AP duration and upstroke velocity can be measured with both electrodes and OM. However, while electrodes are superior to OM in capturing single traces, they are limited when describing the spread of electrical impulses and measurements of conduction velocity that can easily be provided by OM [198,199]. The advent of OM was an important improvement in both cardiac and neural electrophysiology studies [200,201,202].

### 3.2. Application of OM

OM has been applied in embryonic electrophysiology research since the early 80s by Dr. Kamino and his group [35,203,204]. Due to its noninvasiveness, high spatial resolution, and large field of view, OM is ideal for imaging the fragile embryonic heart [197,205]. It was through OM that conduction patterns of the avian embryonic heart and the timing of transitions were confirmed and detailed. The use of OM has been essential in answering basic questions regarding early heart development. A few key studies are summarized below. 

#### 3.2.1. Pioneering OM Studies to Detect Initial Activation Patterns

Kamino and his colleagues were the first to use OM on early avian embryos to study the development of the nervous system and the heart using photodiode arrays [21,142,205]. Conventional techniques such as the use of micro-electrode methods had proven impractical for analyzing the pattern of conduction over the whole heart [142,145]. In the early 1980s Kamino′s group showed spontaneous absorption signals in the immature cardiac muscle (7–9 somites, HH stage 9–10 in the chick embryo). The signal size indicated spontaneous electrical activity in these cells, what we know today as cardiac APs [21].

#### 3.2.2. Finding the Origin of Pacemaker Cells

**Figure 6:**
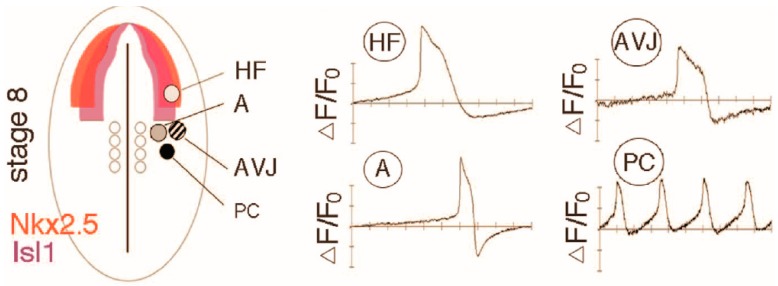
The origin of the pacemaking cells outside the primary and secondary heart fields [33]. Mesoderm cells were isolated from sites indicated by circles on the chicken embryo and cultured. Regions of Nkx2.5 and Isl1 expression are indicated in colors. HF = heart field, A = atrium, AVJ = atrioventricular junction, PC = pacemaking cells. Representative optical tracings obtained by OM of membrane potentials from regions indicated in the diagram after 72 h of culture.

Chicken embryos were studied by OM to identify the origin of pacemaker cells [33]. By precise cell labeling and tracing and OM analysis of the chicken embryo, a novel finding was supported; Pacemaking cells come from a different heart field than the rest of the heart (Figure 6). Lineage studies combined with functional OM studies indicated that cells that are responsible for the first APs in the embryonic heart do not become pacemaking cells that contribute to the pacemaking site in the later stage embryos or the mature sinoatrial node. In addition these latter pacemaker cells originate from mesoderm outside the primary and secondary heart fields and Wnt signaling was identified as being involved in their differentiation [33].

#### 3.2.3. Gradual Transitions in Conduction Patterns

OM studies of the avian heart have confirmed and refined our understanding of the pattern of conduction at stages prior to and after ventricular septation [23,157,158,206,207]. In chicken embryo hearts, this transition was first reported to occur between HH stages 28 and 32 and discovered by the use of electrodes placed at the apex and the base [14] and subsequently by the use of OM [157,159,197,208]. OM further clarified the pattern during the transition using the “breakthrough angle” in the ventricle (Figure 5). The “breakthrough” is the region first activated in the ventricle as observed from the ventral surface of the heart. The breakthrough region moved from the base to the left lateral side of the left ventricle to the right ventricular margin to the apex. The breakthrough at the right ventricular margin is proposed to be the result of the conduction through the interventricular septum and then through the moderator band. The isochrone maps of these stages revealed that the transition from the base-to-apex to apex-to-base pattern of conduction occurred gradually with intermediates stages (Figure 5.).

#### 3.2.4. Heart Field Contributions to Ventricular Maturation

The advantages of the zebrafish model and OM were used to identify the contributions of the first/primary and secondary heart fields to the electrophysiological gradients within the ventricle [209]. The transitions of the tubular heart to become a chambered structure with resultant sequential activation/contraction of the chambers had been followed using electrodes as well as OM. A subsequent step, the electrophysiological maturation of the different regions of the ventricular chamber was demonstrated using OM in the zebrafish heart that develops only a single ventricular chamber. Velocity gradients and vector field maps created using OM data indicated a requirement for the correct ratio of first and second heart field myocardial derivatives to create the complexity of conduction patterns within the single ventricle that control the efficient contraction pattern. This maturation likely precedes the structural septation of the right and left ventricle in other species.

OM also revealed that non-canonical Wnt11/Ca++ signaling plays a role in regulating the electrical gradients within the zebrafish ventricle. Disruption of this Wnt11 signaling in the developing heart resulted in a disappearance of the gradient in conduction velocity between the outer and inner curvature of the ventricle [210].

#### 3.2.5. Following the Conduction of the Atrioventricular Junction

**Figure 7:**
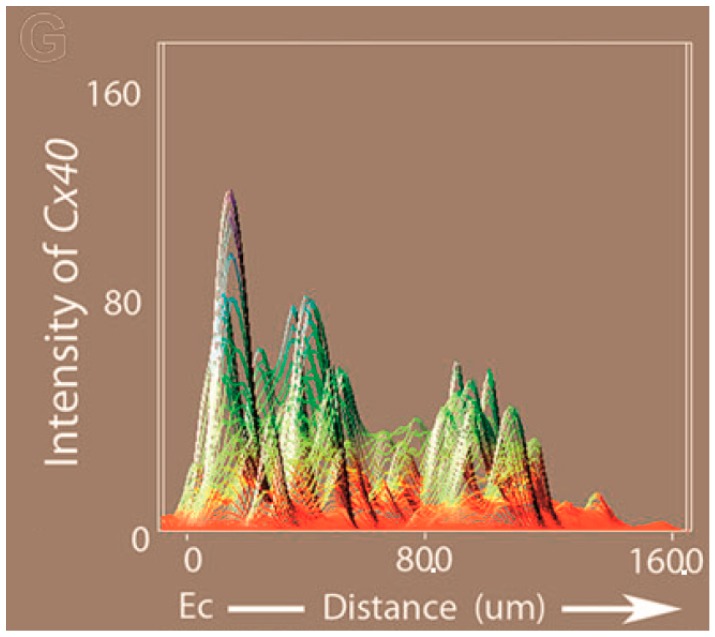
Proximity to the endocardium regulates Cx40 expression in atrioventricular junction (AVJ) myocytes [43]. Aggregates of AVJ cardiomyocytes were microsurgically implanted into the host superior endocardial cushions of HH stage 15–16 chicken embryos at different distances in microns from the endocardium. After 14 h of culture, the maximal intensity of Cx40 expression in the aggregates was determined by *in situ* hybridization and compared to their distance from the endocardium (Ec). The closer to the endocardium, the higher the intensity of Cx40 expression.

It has long been known from studies using electrodes that the distinct properties of the AVJ emerge during early chamber formation [38,39] distinguishing it from flanking regions, the atrial and ventricular myocardium. Originally it was proposed that the slower conducting AVJ retains the characteristics of the slow conducting “primitive” heart tube [38,41,45]. Recent findings using OM technology support a more complex hypothesis. The AVJ at the looping stage does not seem to be simply retaining its “primitive” heart tube properties because the AP morphology of these two myocardia differ in conduction velocity, AP upstroke velocity and AP duration [33]. This same study found data (Figure 7) consistent with the hypothesis that differential growth of the endocardial cushions at the AVJ results in reduced endothelin 1 (Et1) signaling between the endocardium and the myocardium [33]. This reduced signaling may be due to the increased distance that signaling factors secreted by endocardial cells must travel to reach the myocardium. Meanwhile, the flanking regions, the atria and ventricles receive more Et1 signaling because the cardiac jelly between the endocardium to myocardium is very thin and that boosts the expression of Cx40 in the myocardium. The outcome is a lower level of gap junction Cx40 expression in the AVJ than in the flanking regions. Because Cx40 is associated with fast conduction, this may account in part for the resulting alternating regions of fast and slow conduction that emerges at the looping heart stages (Figure 3).

#### 3.2.6. Later Maturation of the Conduction System

OM was also used to show that the cardiac neural crest cells (CNCCs) are playing a role in late maturation of the cardiac conduction system [159]. Labeled neural crest cells in chicken and mouse models revealed that these special cells travel to venous as well as the arterial poles of the heart entering the dorsal aspect of the heart and traveling across the crest of the primitive IVS to surround the central conduction system [56]. Ablation of the cardiac neural crest (from the level of the otic placode to somite 3) resulted in abnormal morphology of the ventricular conduction system as well as in the conduction pattern of the chicken heart. The common bundle failed to become compact remaining large in diameter and “shaggy”, that is, apparently maintaining connections to the surrounding myocardium. The conduction pattern derived from OM data indicated that the segmenting heart was not achieving the mature apex-to-base conduction pattern. Cardiac neural crest ablated embryos exhibited breakthroughs in conduction at the base of the heart at a stage post-septation when none were evident at that site in the unablated controls of the same stage. Authors suggested that the separation and insulation of the cardiac conduction system required the presence of cardiac neural crest cells. How the CNCCs accomplish that is unknown. Possibilities include that they secrete the extracellular matrix surrounding the conduction system.

### 3.3. Current Limitations of OM and Potential Solutions

As powerful as OM is for delving into the electrophysiology of the embryonic heart, there are several limitations of OM that require the development of new technologies to overcome.

#### 3.3.1. OM Is a Terminal Procedure

One limitation of OM is that it is a terminal procedure. Current protocols require removal of the heart from the embryo to immerse into motion suppression drugs and the voltage sensitive dyes that are both toxic. With current detection methods, contraction of the heart has to be stopped to prevent motion artifact. With a motion free heart, high quality AP traces can be acquired. The compounds commonly used to prevent contraction by different mechanisms are 2,3-butanedione monoxine, cytochalasin D and blebbistatin. They have been shown to maintain electrical propagation but may alter the electrophysiology of the heart or otherwise cause artifacts and are eventually toxic [211,212]. Obviously, preventing contraction also eliminates the possibility of analyzing the interesting relationship between excitation and contraction during cardiac development.

Studies have been performed on mouse embryo hearts without using motion suppression reagents with results inconsistent with studies on avian embryos studied after exposure to motion suppression drugs [102,213]. It is still not clear if this difference in conduction patterns between mouse and chicken embryo pre-septation stage hearts are due to fundamental species differences or due to differences in the protocols used to acquire OM data. An alternative strategy to avoid the use of motion suppression drugs because of their potential negative effects is to remove motion through ratiometric imaging or post processing [195,214,215]. These latter strategies have not been satisfactory and have not been used subsequently.

#### 3.3.2. OM Is a 2D Projection from a 3D Structure

OM collects information from a 3D surface as a 2D projection map [216,217,218]. While the general activation sequence and AP morphology analyses are not affected by projection, ignoring the curvature of the heart surface results in errors when calculating conduction velocity. Curvature is especially pronounced in early stage tubular hearts and can vary greatly at different developmental stages and between individual embryos at similar stages [219]. 

This limitation can be partially overcome by flipping over the heart and mapping both sides, using multiple cameras to take data at different planes simultaneously, or using angled mirrors to collect data from more of the surface at a time. Each of these potential solutions comes with their own difficulties. Flipping the heart can damage the tissue and even if there is no damage, spatiotemporal registration of the data from the two sides that are taken at different times requires special algorithms. Using two cameras is expensive and the data would have to be registered/synchronized. Use of an angled mirror overcomes some of the limitations of OM as a 2D detection method by revealing and allowing recordings from two surfaces of the heart simultaneously thus covering 75% of the surface without handling or moving the heart or adding extra cameras [159]. The soft and sensitive heart tissues at these stages are not amenable to flattening against glass as is done for the mature heart. 

At early stages the surface OM findings are assumed to represent the properties of the thin heart wall. For thicker cardiac tissues encountered at later stages, the electrophysiological properties of the different layers of myocardium may be distinct and require the dissection of tissue as in the wedge preparation [220,221] or by opening up the heart to access the endocardial surface where the main branches of the ventricular conduction system lie [213]. Heart tissue under these conditions require perfusion and temperature control and yet even under the best conditions such as the Langendorff preparation [222] an adult heart would not survive longer than 6-8 h with the standard perfusion of glucose and Ringer′s solution. An exception is an unusual study of adult rabbit hearts perfused with the blood substitute Perftoran [Perfluorocarbon, (223]) that allowed continued beating or several days. Such perfusion is difficult to undertake in a small and fragile embryonic heart.

**Figure 8:**
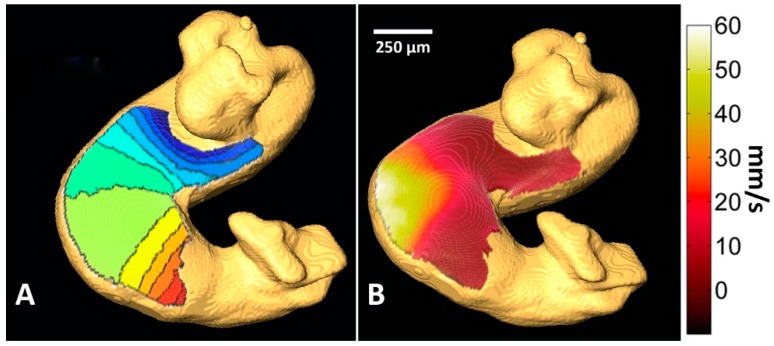
OM data projected onto a 3D surface map [226]. OM data was obtained from HH stage 15 quail embryo hearts. (**A**) The activation isochrome map obtained using data from OM was projected onto a 3-D structural map of the tubular heart obtained by OCT. Activation begins in the atria at the top and the outflow tract at the bottom with the conduction propagating from blue to red (early to late) regions with each line representing a 10-ms interval. The AVJ, mapped in blue, has slower conduction than the ventricle in green. (**B**) Conduction velocity mapped in 3D shows a hot spot of highest conduction velocity (yellow) on the outer curvature of the ventricle. Regions around the atrium and outflow tract were not included in the OM analysis because they were out-of-focus.

2-D OM combined with optical coherence tomography (OCT) was developed to partially solve the issue of the 2-D projection from a 3-D structure [224]. OCT was used to acquire the 3D topology of the heart while the OM signal was collected simultaneously and projected onto it. This allowed calculation of more accurate conduction velocities using 3D surface vectors (Figure 8). In another study, 3D sectioning microscopes (confocal microscopes) have been used to acquire calcium and voltage APs in 3-D from zebrafish hearts across many stages of development and allowed documentation of region-specific transitions in currents [225]. Studies have just begun to be carried out using these novel combinations of technologies to probe the developing heart. 

#### 3.3.3. OM Has a Lower Temporal Resolution than Electrodes

The temporal resolution of OM for a large number of pixels is still much lower than electrode recordings that can easily go higher than 10 kHz in sampling rate [227]. However, the temporal resolution required for embryonic hearts does not exceed 10 kHz even at the later stages. Typical frame rates for OM ranges from 500–2000 Hz [33,210,224]. This limits accurate measurements of fast action potential upstrokes. With the developing speed of high sensitivity CCD cameras (≥500–2000 Hz) the problem has become less critical. The temporal response of the dye is also another factor to consider. The dye has to respond to the voltage changes fast enough to capture quick changes of the membrane potential. This is important for accurately measuring AP morphology, and also for mapping conduction and computing conduction velocity, where accurate detection of the activation time is needed [33,210,224,227].

#### 3.3.4. The Embryonic Heart Has Low Signal to Noise Ratios

**Figure 9:**
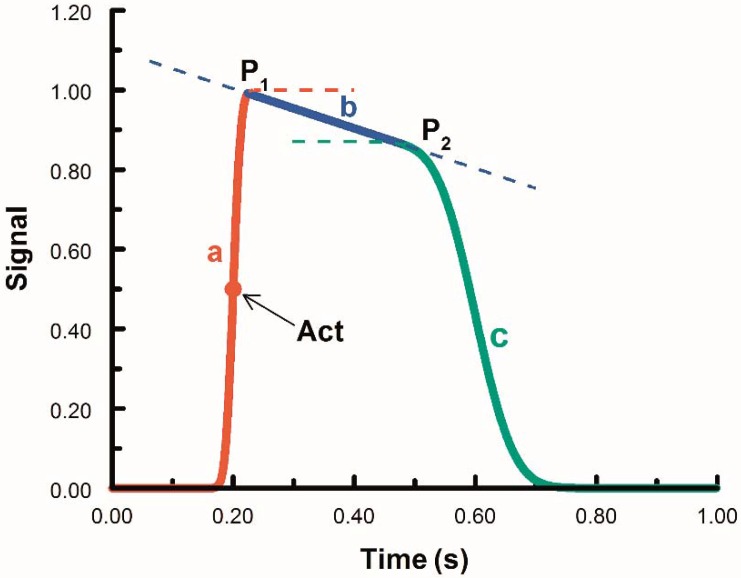
Linear Fit Functions to fit the cardiac APs from OM data [226]. Curve a (red) and c (green) are cumulative normal distributions, line b (blue) is a linear function. P1 and P2 are junction points connecting the three functions. Together, the solid lines for a, b, and c comprise the fitted AP. The red dot is the time of activation (arrow, Act), which is 50% of the maximum value of the first cumulative normal.

OM in embryonic hearts, especially younger embryonic hearts, usually suffers from low signal to noise ratios (SNRs) because of relatively small and integrating signals from very few cells. For example, Di-4-ANEPPS, a popular voltage sensitive dye used for OM analysis of embryonic hearts, provides a typical relative change in fluorescence (ΔF/F) of 1%–5% under optimal filter settings. Embryonic hearts at looping stages are only two cell layers [2,228]. Under a high resolution OM microscope, only a few cells contribute a small signal to a pixel with the background of multiple noise sources. The resultant SNR may range from 2–10 with the center of the heart typically under 5 and the edge of the heart above 5 (pixels at the edge of the heart collect signals from more cells) without averaging. The low SNR would cause difficulty and inaccuracy in electrophysiology measurements. High-sensitivity, low-noise EMCCD camera can partially solve this problem. Increased temporal resolution can also provide more accurate measurement of activation time from an AP [226]. The development of more robust algorithms for data analysis in low SNR environments can be very effective (Figure 9). For example, a piece-wise fit of the measured, noisy AP trace to a pair of cumulative normal functions bridged by a line enabled more accurate detection of the activation time. Furthermore, a 2D linear fit is much more precise in calculating the conduction velocity than the central difference method or the Sobel filter [226]. 

The development of new and better voltage sensitive dyes can improve Δ F/F and potentially improve SNR significantly. A recently commercially available dye FluoVolt^™^ (FluroVolt^™^ membrane potential kit from Molecular Probes^™^, division of Thermo Fisher Scientific, Eugene, Oregon) provides more than a 5 times SNR improvement over Di-4-ANEPPS in OM of quail embryonic hearts (Figure 4). This dye has been used to sensitively analyze iPS cell-derived cardiomyocytes in culture simultaneously with the use of multi-electrode arrays [229].

### 3.4. Genetic Reporters

Voltage- and calcium-sensitive dyes have several disadvantages including limited live animal studies, increased toxicity, the need to load the cells, and limited cell selectivity. These disadvantages may be overcome by genetically engineered alternatives. Recently, genetic reporters for calcium and voltage changes have opened up new opportunities for studying electrophysiology in the embryonic heart. The development of voltage sensitive fluorescent protein markers is progressing rapidly and may, in combination with imaging systems, capture heart development function in 3-D longitudinally [230].

Chi *et al.* demonstrated a genetic calcium indicator based on gCaMP and studied four distinct stages of cardiac conduction development in the zebrafish heart [80]. Since this initial report, gCaMP has gone through several iterations to increase the fluorescent signal and temporal dynamics. GCaMP6 is the latest version of the calcium indicator [231] and a reporter mouse with a Thy1 promoter is available at Jackson Labs [232]. The Thy1 promoter allows expression in neurons. Kralj *et al.* demonstrated an archaerhodopsin-based genetic voltage indicator in bacteria [233] and have since developed brighter archaerhodopsin-based indicators that can be combined with channel rhodopsins for perturbation and measurement [234] or calcium indicators [225]. Recently, Gong *et al.*, fused *Acetabularia acetabulum* rhodopsin with mNeon-Green to enable bright, fast voltage responses (<1 ms responses) in live mice and flies [235]. These reporters open up exciting possibilities for studying the developing conduction system.

## 4. Controlling the Electrophysiology of the Heart 

### 4.1. The Disadvantages of Electrode Stimulation

In the clinic, point stimulation using electrodes on the heart is routinely utilized to diagnose and treat arrhythmias because many electroPhysiologic parameters are rate dependent (e.g., conduction velocity). However electrical pacing has several disadvantages that make it unsuitable for point stimulation in early embryonic heart studies. First, electrode stimulation excites an area much larger than the electrode tip, which makes point stimulation extremely difficult if not impossible in embryonic hearts [236]. Second, injecting current into the tissue causes electrical millimeter scale artifact that can significantly obscure electrical recordings in small embryonic hearts [237,238,239]. This scale of artifact would greatly affect recordings from an E8.5 mouse embryo heart (looping heart pre-septation) which is less than 1 mm in length. Third, high charge densities needed for electrical point stimulation of small tissues can lead to damage [240]. Finally, the electrode must be in contact with the tissue, which leads to added experimental difficulties. Positioning the electrode for contact on a beating heart takes extra time with an increased risk of damaging the fragile tissue of the embryonic hearts with the electrode tip.

### 4.2. Infrared Pacing and Optogenetics

**Figure 10:**
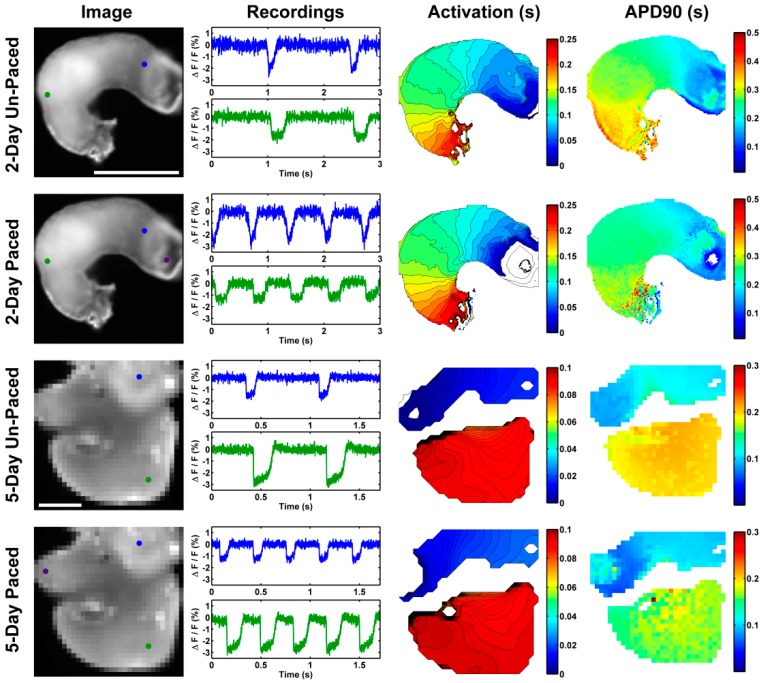
Optical pacing combined with optical mapping. Representative data from 2-day (HH stage 15), with no binning (**A**,**B**), and 5-day (HH stage 27), with 4 × 4 binning (**C**,**D**), excised embryonic quail hearts. Fluorescence images of the hearts (Image) have a blue dot on the atrioventricular junction (2 day) or atrium (5 day) and a green dot on the ventricle. The purple dot indicates the position of the pacing laser (**B**,**D**). Scale bars are 500 µm. Representative recordings of electrical activity are shown for the matching colored markers. Activation maps (Activations(s)) are shown with the color map in seconds and the isochrones are 10 ms apart for the 2-day heart (**A**,**B**) and 1 ms apart for the 5-day heart (**C**,**D**). Action potential duration maps [APD90 (s)] are shown with the color map in seconds [245].

Optical approaches are an attractive alternative to electrical pacing for point stimulation in small tissues. Jenkins *et al.* first demonstrated the capability of light to induce cardiac pacing [241]. Briefly, a pulsed infrared laser (1875 nm) induced a thermal gradient in the tissue causing the heart to contract after the delivery of each pulse (Figure 10). Functional assays and transmission electron microscopy showed no evidence of tissue or cell damage. Subsequently, two papers reported on the use of cardiac optogenetics [242,243]. Bruegmann *et al.* expressed channelrhodopsin-2 in mice hearts, while Arrenberg *et al.* expressed halorhodopsin and channelrhodopsin in zebrafish cardiomyocytes. Channelrhodopsin-2 enabled optical pacing with pulsed blue light, while halorhodopsin enabled light-activated cessation of the heartbeat with orange light. Both optogenetic studies showed reversibility of their interventions and no signs of light damage to the tissue. Since these initial studies, infrared pacing has been applied to neonatal rabbit cardiomyocytes [244], avian embryo hearts [224,226,245], mouse embryo hearts [245] and adult rabbit hearts [246]; while optogenetic pacing has been applied in human cardiac cells [247], rat cardiac cells [248,249,250,251], guinea pig cardiac cells [252], and *Drosophila melanogaster* hearts [70].

Both infrared pacing [245] and optogenetic pacing [243] can induce arrhythmias such as tachycardia and 2 to 1 atrioventricular block. Recent studies have even shown the ability of optogenetic pacing to create [251] and terminate [249] spiral waves, which manifest in reentrant arrhythmias. The advantages of infrared pacing include no need to introduce exogenous genes and no overlap of the stimulation light with common fluorescent reporters. The advantages of optogenetic pacing include no heating of the tissue and a clearer understanding of the mechanism for activation. Both techniques overcome the limitations of electrical pacing; both can be delivered with high spatial precision. Both are reversible with no obvious tissue damage; neither produces an electrical artifact; neither induces high charge densities; neither requires contact with the tissue during activation.

## 5. Summary and Future Directions

Recent developments in technology enable detailed and quantitative studies of the origin and differentiation of the pacemaking and conduction system in culture and *in vivo*. Still barriers remain to be overcome. First, techniques allowing simultaneous measurement of contraction and conduction dynamics are needed. Abnormal electromechanical coupling likely contributes to several disease processes, but because of limitations in technology very little is known. Motion correction algorithms or multimodality imaging may enable these studies in the future. Second, *in vivo* imaging of cardiac electrophysiology would enable greater insight into abnormal/normal development of the conduction system. The quickly advancing field of genetically encoded calcium/voltage indicators holds the promise of moving away from phototoxic dyes and enabling live imaging in intact embryos. Third, robust methods for point stimulation in embryos to assess rate-dependent electrophysiology parameters may finally be addressed with the recent advancements in optical pacing with either infrared light or optogenetics. Fourth, longitudinal imaging is needed to follow the evolution of the conduction system. Finally, electrophysiologic data must be integrated with structural and molecular data to determine the etiology of arrhythmias in the pediatric population. Correlating changes in molecular expression and structure with the functional data would results in a more complete picture of all the factors that interact to control cardiac development. Although much work needs to be done, these goals are now within reach [230,253,254].

## Figures and Tables

**Table 1:** Mouse lines for the study of the developing pacemaking and cardiac conduction system.

Mouse Line	Other Names (See MGI)	References
CCS-lacZ	Tg(En2-lacZ)1Alj, MC4	Rentschler *et al.*, 2001 [102]
Cx30.2-LacZ	Gjd3*tm1.2Kwi*, Cx30.2*LacZ*	Kreuzberge *et al.*, 2005 [104]
Cx45-LacZ	Gjc1*tm1Kwi*	Kreuzberg *et al.*, 2005 [104] and Kruger *et al.*, 2000 [105]
HCN4-CreERT2	Hcn4*tm1(cre-/ERTs)Anlu or* Hcn4*tm1(cre/ESR1)Anlu*, Hcn4*tm2.1(cre/ERT2)Sev*	Hoesl *et al.*, 2008 [106], Spater *et al.*, 2013 [107] derived from Liang *et al.*, 2013 [108]
Hcn4-CreERT2	Tg(Hcn4-cre/ERT2)1Yzhao	Wu *et al.*, 2014 [109]
HCN4-nEGFP	Hcn4*tm1Sev*, HCN4-H2B-EGFP	Liang *et al.*, 2013 [108], Sun *et al.*, 2007 [110]
HCN4-nLacZ	HCN4H2BGFP HCN4nlacZ (both markers in one)	Liang *et al.*, 2013 [108]

These mouse lines have been shown to express markers for most of the pacemaking and CCS from early heart development and in the adult. For a complete list of more mouse lines see Liang *et al.*, 2015. MGI = Mouse Genome Informatics (http://www.informatics.jax.org/).
